# Satisfaction Survey of Women After Cosmetic Genital Procedures: A Cross-Sectional Study From Saudi Arabia

**DOI:** 10.1093/asjof/ojaa048

**Published:** 2020-11-10

**Authors:** Modhi M Al-Jumah, Shorug K Al-Wailiy, Ahmed Al-Badr

**Affiliations:** College of Medicine, AlFarabi Colleges, Riyadh, Saudi Arabia; College of Medicine, AlFarabi Colleges, Riyadh, Saudi Arabia; Department of Urogynecology & Pelvic Reconstruction Surgery, Women’s Specialized Hospital, King Fahad Medical City, Riyadh, Saudi Arabia

## Abstract

**Background:**

Female cosmetic genital surgery (FCGS) aims for better aesthetic genital appearance and improved functional aspects to enhance women’s self-esteem and satisfaction.

**Objectives:**

This study aims to assess the satisfaction of women who have undergone FCGS and its impact on their sexual, psychological, and aesthetic aspects.

**Methods:**

An observational cross-sectional study was conducted in private clinics in Riyadh, Kingdom of Saudi Arabia, between March and June 2019, in women who underwent FCGS. Phone interviews were conducted in the Arabic language. The survey comprised 4 sections: demographics, motives for FCGS, quality-of-life questionnaires about genital appearance satisfaction, and sexual function.

**Results:**

Out of the 196 women undergoing FCGS during the study period, 11.7% refused to participate, and 37.2% did not answer phone calls; 51% of the women participated in the study. The women’s age ranged between 23 and 55 years; 64% underwent vaginoplasty, and 73% underwent other cosmetic procedures. Ninety-two percent of the women did not have any complications after these procedures.

**Conclusions:**

In this group of women, FCGS was safe and effective, and the majority of participants reported overall satisfaction and improvement of sexual function, genital appearance, and self-esteem.

**Level of Evidence: 4:**

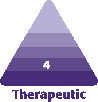

Female cosmetic genital surgery (FCGS) aims for better aesthetic genital appearance and improved functional aspects. Although numerous procedures fall under FCGS, one of the most common FCGS is labiaplasty, which includes the reduction or augmentation of the labia minora or labia majora. These surgeries are performed either by gynecologists or by plastic surgeons. Labiaplasty changes the size or shape of the labia, typically making them smaller or correcting an asymmetry between the 2 sides. Reduction labiaplasty can be performed using several techniques such as the cold cutting technique, which uses an energy-based device (laser or radiofrequency generator) or the electrosurgical cutting technique. Another common procedure is vaginal rejuvenation, which encompasses perineoplasty and vaginoplasty, and these are performed mainly by gynecologists.^1^

Evidence suggests an increasing trend for requests for FCGS derived from the women’s desire for a standardized genital appearance and function termed as the “Barbie doll look.” In this look, the labia minora are narrow and invisible, and the vaginal opening appears very tight; this genital appearance is the new ideal of the “perfect vagina” ^2^ among women. In recent years, FCGS procedures have become popular, with a drastic increase in demand in Western countries; FCGS has also gained popularity in Gulf countries. It has been reported that in the United States, requests for FCGS increased from 5070 in 2013 to 7535 in 2014 (49% increase).^3^

According to Sharp et al,^3^ the influencing factors that led women to undergo labiaplasty were aesthetic dissatisfaction with labia in 87%, discomfort when wearing clothing in 64%, painful sexual intercourse in 43%, and discomfort when taking part in sports in 26% of the women. The leading cause of labial hypertrophy is unknown.^4^ However, it can be congenital, or can occur after multiple pregnancies, due to stretching; depending on asymmetry due to excessive tissue protrusion, labial hypertrophy is classified as type I (<2 cm), type II (2-4 cm), type III (4-6 cm), or type IV (>6 cm).^5^ Although there is no ideal surgical technique for labiaplasty, each technique has pros and cons. Standard labial reduction procedures include either elliptical excision or wedge excision.^5,6^

This study aims to assess the satisfaction of women who have undergone FCGS labiaplasty or vaginoplasty and the impact of FCGS on the measures of sexual functioning, psychological well-being, and aesthetics.

## METHODS

An observational cross-sectional study was conducted to assess the outcome and satisfaction rate of women who underwent FCGS in 2 private clinics in Riyadh, Kingdom of Saudi Arabia, between January 2016 and January 2019. All FCGSs were performed by the same senior surgeon (A.H.B.), who has been practicing for 15 years. 

The study recruited all women who underwent FCGS labiaplasty (either labia majora reduction or labia minora reduction), vaginoplasty, or any other cosmetic procedure such as augmentation of labia majora by filler or liposuction and fat transfer.

Inclusion criteria were as follows: Saudi nationality, aged between 18 and 55 years, and women who underwent cosmetic genital procedures within the period from 2010 to 2019 with the same surgeon (A.H.B.). Women who were not willing to participate in the phone interview were excluded. 

Data were collected between March and June 2019. All candidates were called and asked to participate. The objectives and benefits of the study were explained in brief to the participants, and verbal consent was obtained before the questionnaire survey (Supplementary Appendices A and B). Data were collected using phone interviews by 2 of the researchers (M.M.J and S.K.W., medical students); interviews were conducted in the Arabic language to enhance the participants’ understanding. Eligible women were called up to 4 times to complete the survey. Medical charts in the clinic were reviewed for demographics, types of procedures, dates, and any recorded complications. In addition, an extensive literature review was performed to establish and develop our survey, and the survey was created to match and cover the study objectives. All participants completed the questionnaire comprising of 4 sections:

1. *Demographics*: Participants were asked about their age, body mass index (BMI; height and weight), parity, and educational status.
2. *Motives for FCGS and Quality of Life*: Participants were asked about the date of surgery, the time of the follow-up appointment, and open-ended questions regarding the reasons for having the surgery. Responses were classified into appearance, functional, sexual, and psychological categories.
3. *Genital Appearance Satisfaction*: Participants’ satisfaction with their genital appearance was measured on the genital appearance satisfaction scale, using a validated 11-item questionnaire.^7^ This questionnaire encompassed 3 factors, as follows: “appearance of genitals,” “impact on daily living,” and “impact on sex.” The comparison between parameters before and after procedures was based on the normal appearance, attractiveness, and symmetry of genitals, satisfaction rate, psychological effect, discomfort or irritation during activities, embarrassment during sexual intercourse, and feelings of worry regarding labia being too large, or being visible when wearing tight clothes.
4. *Sexual Function*: Participants were asked about sexual function before and after the procedure, by using 6 items picked from the validated Arabic quality of life (QoL) version of the Pelvic/Urinary Incontinence Sexual Questionnaire (PISQ); this questionnaire comprises of questions on how often the participant felt aroused, fulfilled, ashamed, fearful, or in pain during sexual intercourse, and finally, the level of sexual desire before and after the procedure.^8^

Data were entered and analyzed using the SPSS 25.0 software (SPSS Inc., Chicago, IL). Demographic characteristics of study participants were reported as mean (standard deviation [SD]) and median (minimum and maximum) for continuous variables. Categorical variables were reported as counts (percentage). Categorical data for assessing differences in the proportion of patients in agreement with individual items before and after surgery were analyzed using the McNamara’s test of marginal homogeneity. This test evaluates the significance of the difference in categorical responses in repeated measurements (before vs after an intervention) in a sample. A 2-tailed *P*-value of 0.05 was considered significant.

The study was reviewed and approved on January 31, 2019 by the Institutional Review Board (IRB) of King Fahad Medical City, Riyadh, Saudi Arabia (IRB 19-072).

## RESULTS

A total of 196 women underwent FCGS between January 2016 and January 2019. Among them, 100 (51%) women were included in the study, as 23 (11.7%) women refused to participate, and 73 (37.2%) women did not respond to repeated calls. The age of the study participants ranged from 23 to 55 years (mean [SD] 36.9 [7.7] years). BMI of most of the participants (38%) was normal (BMI 18-24) (mean [SD] 149.7 lbs [26.4]). Forty-three women who sought the FCGS had 4 or more children, while 10 never had any children. Seventy-five percent of the participants had university level (63%) or a higher level of educational (12%) (Table 1).

**Table 1.** Demographics

Parameter	Mean	Standard Deviation (*N*) %
Age (23-55 years)	36.88	7.65
Body mass index	Underweight	(0) 0.0%
	Normal	(38) 38.0%
	Overweight	(37) 37.0%
	Obese	(25) 25.0%
Number of children	No children	(10) 10.0%
	One child	(12)12.0%
	Two children	(9) 9.0%
	Three children	(26) 26.0%
	Four or more children	(43) 43.0%
Education level	School level	(25) 25.0%
	University level	(63) 63.0%
	Masters/PhD	(12) 12.0%

PhD, Doctor of Philosophy.

Various procedures were performed, including vaginoplasty in 64%, labiaplasty (labia minora reduction) in 47%, augmentation of labia majora by filler in 13%, labiaplasty “labia majora reduction” in 10%, and liposuction plus fat transfer in 3% of the women. Some of the women (28%) underwent multiple combined procedures. Thirteen women underwent FCGS in 2016, 49 in 2017, 32 in 2018, and 6 in 2019.

The period between procedure date and the call date ranged from 1 to 36 months (mean [SD] 19.95 [9.68] months).

**Figure 1.**
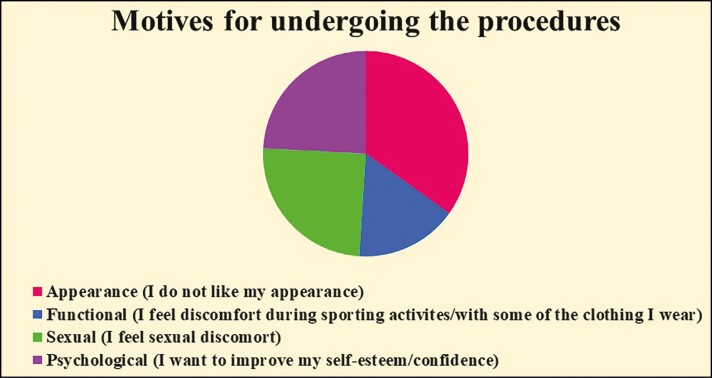
Motives for undergoing the procedures.

Figure 1 shows the main reasons for undergoing the procedures. Approximately, 52% of women did not like the appearance of their genitals, 38% had sexual discomfort, 37% wanted to improve self-esteem/confidence, while 24% had trouble during sporting activities/with clothing. 

Ninety-two percent of the women did not have any complications after the procedures, while 8% encountered complications, such as pain (2%), urinary tract infection (5%), and delayed wound healing (1%). However, none of the participants needed a repeat surgery.

Genital appearance satisfaction scores before and after the procedures are provided in Table 2. A comparison was made between women who underwent vaginoplasty and those who underwent other cosmetic surgery procedures. The perception of normal appearance of genitals was increased by 35.5% in women after the vaginoplasty procedure, while after other cosmetic procedures, this feeling was increased by 55.5%. A perception of the genitals being unattractive was decreased by 57.8% after vaginoplasty and decreased by 61.1% after other cosmetic procedures. The feeling of “labia enlargement” was reduced by 58.3% in women after performing other cosmetic procedures, while it decreased by 29.7% after implementing vaginoplasty. The satisfaction rate increased by around 50% in both groups. A 40% improvement (approximately) in psychological parameters was reported after the procedures in both groups; self-confidence and self-esteem were increased as well. Feelings of discomfort or irritation during activities or when wearing tight clothes improved by approximately 35% after the procedures in both groups; the feeling of genitals being visible with tight clothes decreased by 33.3% after other cosmetic procedures and by 18% after vaginoplasty. Moreover, feelings of embarrassment during sexual intercourse decreased by 50% after vaginoplasty and by 36.1% after other cosmetic procedures. Feeling worried about the appearance of the genitals decreased by approximately 34.3% after vaginoplasty and by 44.4% after other cosmetic procedures. Approximately, 63% of women were happy and satisfied after vaginoplasty, while 31% were satisfied after other cosmetic procedures. The asymmetric look of genitals was decreased by approximately 40% after the procedures in both groups. Overall, 90% of cases reported significant improvement in the appearance of the genitals. Psychological parameters, self-confidence, and self-esteem increased by approximately 40% after vaginoplasty. Furthermore, a significant decrease was reported in the following: discomfort or irritation during activities or with tight clothes and embarrassment during sexual intercourse.

**Table 2.** The Genital Appearance Satisfaction Parameters: Comparison of Satisfaction Rate Before and After Undergoing Cosmetic Vaginal Procedures

		Vaginoplasty			Other Cosmetic Procedures		
		Before *N* (%)	After *N* (%)	*P*-value	Before *N* (%)	After *N* (%)	*P*-value
Feel the genitals are normal in appearance	Agree	37 (57.8%)	61 (95.3%)	<0.0001	14 (38.9%)	34 (94.4%)	<0.0001
	Disagree	7 (42.2%)	3 (4.7%)	<0.0001	22 (61.1%)	2 (5.6%)	<0.0001
Feel the genitals are unattractive	Agree	40 (62.5%)	3 (4.7%)	<0.0001	27 (75.0%)	5 (13.9%)	<0.0001
	Disagree	24 (37.5%)	61 (95.3%)	<0.0001	9 (25.0%)	31 (86.1%)	<0.0001
Feel the labia are too large	Agree	27 (42.2%)	8 (12.5%)	<0.0001	26 (72.2%)	5 (13.9%)	<0.0001
	Disagree	37 (57.8%)	56 (87.5%)	<0.0001	10 (27.8%)	31 (86.1%)	<0.0001
Satisfaction rate	Satisfied	26 (40.6%)	57 (89.1%)	<0.0001	9 (25.0%)	32 (88.9%)	<0.0001
	Unsatisfied	38 (59.4%)	7 (10.9%)	<0.0001	27 (75.0%)	4 (11.1%)	<0.0001
Psychological effect	Satisfied	28 (43.8%)	57 (89.1%)	<0.0001	19 (52.8%)	31 (86.1%)	0.004
	Unsatisfied	36 (56.3%)	7 (10.9%)	<0.0001	17 (47.2%)	5 (13.9%)	0.004
Self-confidence and self-esteem	Satisfied	27 (42.2%)	57 (89.1%)	<0.0001	16 (44.4%)	32 (88.9%)	0.001
	Unsatisfied	37 (57.8%)	7 (10.9%)	<0.0001	20 (55.6%)	4 (11.1%)	0.001
Feel irritation and discomfort during activities	Yes	22 (34.4%)	1 (1.6%)	<0.0001	14 (38.9%)	2 (5.6%)	0.004
	No	42 (65.6%)	63 (98.4%)	<0.0001	22 (61.1%)	34 (94.4%)	0.004
Feel embarrassment during sexual intercourse	Yes	37 (57.8%)	5 (7.8%)	<0.0001	22 (61.1%)	9 (25.0%)	0.011
	No	27 (42.2%)	59 (92.2%)	<0.0001	14 (38.9%)	27 (75.0%)	0.011
Feel uncomfortable wearing tight clothes	Yes	18 (28.1%)	3 (4.7%)	<0.0001	17 (47.2%)	2 (5.6%)	0.001
	No	46 (71.9%)	61 (95.3%)	<0.0001	19 (52.8%)	34 (94.4%)	0.001
Feel genital area is visible with tight clothes	Yes	18 (28.1%)	7 (10.7%)	0.013	17 (47.2%)	5 (13.9%)	0.01
	No	46 (71.9%)	57 (89.1%)	0.013	19 (52.8%)	31 (86.1%)	0.01
Feel worry about the appearance of genitals	Yes	23 (35.9%)	1 (1.6%)	<0.0001	21 (58.3%)	5 (13.9%)	<0.0001
	No	41 (64.1%)	63 (98.4%)	<0.0001	15 (41.7%)	31 (86.1%)	<0.0001
Feel genital area looks asymmetric	Yes	34 (53.1%)	7 (10.9%)	<0.0001	25 (69.4%)	11 (30.6%)	0.01
	No	30 (46.9%)	57 (89.1%)	<0.0001	11 (30.6%)	25 (69.4%)	0.01

Table 3 details the sexual function and QoL parameters before and after the procedures. For vaginoplasty, feeling aroused during sexual intercourse was reported as “rarely or never” before the procedure in 56.2% of the women and in 21.9% after the procedure. Feeling fulfilled during the sexual intercourse was reported as “rarely or never” before the procedure in 82.8% and in 21.9% after the procedure. Feeling shame rarely or never during sexual intercourse was reported by 37.5% of women before the procedure and by 92% after the procedure. Feeling fear during the sexual intercourse was reported as “rarely or never” before vaginoplasty in 15.6% and after vaginoplasty in 90.6% of the women. The incidence of “very high or high” level of sexual desire increased from 14.1% before the procedure to 40.6% after the procedure. Feeling pain during the sexual intercourse was reported as “rarely or never” by 96.9% before vaginoplasty and by 37.5% of women after vaginoplasty.

**Table 3.** Sexual Function Comparison Before and After Procedures

		Vaginoplasty		Other Cosmetic Procedures	
		Before Count (Total, *N* %)	After Count (Total, *N* %)	Before Count (Total, *N* %)	After Count (Total, *N* %)
How often do you feel aroused during sexual activity?	Never	7 (10.9%)	11 (17.2%)	8 (22.2%)	9 (25.0%)
	Rarely	29 (45.3%)	3 (4.7%)	16 (44.4%)	3 (8.3%)
	Sometimes	16 (25.0%)	19 (29.7%)	6 (16.7%)	9 (25.0%)
	Usually	9 (14.1%)	19 (29.7%)	4 (16.7%)	10 (27.8%)
	Always	3 (4.7%)	12 (18.8%)	2 (5.6%)	5 (13.9%)
When you engage in sexual activity do you feel fulfilled?	Never	22 (34.4%)	9 (14.1%)	15 (41.7%)	11 (30.6%)
	Rarely	31 (48.4%)	5 (7.8%)	16 (44.4%)	2 (5.6%)
	Sometimes	11 (17.2%)	14 (21.9%)	5 (13.9%)	11 (30.6%)
	Usually	0 (0.0%)	21 (32.8%)	0 (0.0%)	8 (22.2%)
	Always	0 (0.0%)	15 (23.4%)	0 (0.0%)	4 (11.1%)
When you engage in sexual activity do you feel shame?	Never	0 (0.0%)	52 (81.3%)	0 (0.0%)	27 (75.0%)
	Rarely	24 (37.5%)	7 (10.9%)	4 (11.1%)	2 (5.6%)
	Sometimes	15 (23.4%)	2 (3.1%)	8 (22.2%)	3 (8.3%)
	Usually	12 (18.8%)	0 (0.0%)	17 (47.2%)	0 (0.0%)
	Always	13 (20.3%)	3 (4.7%)	7 (19.4%)	4 (11.1%)
When you engage in sexual activity do you feel fear?	Never	3 (4.7%)	55 (85.9%)	2 (5.6%)	32 (88.9%)
	Rarely	7 (10.9%)	3 (4.7%)	5 (13.9%)	0 (0.0%)
	Sometimes	17 (26.6%)	1 (1.6%)	5 (13.9%)	2 (5.6%)
	Usually	9 (14.1%)	1 (1.6%)	13 (36.1%)	1 (2.8%)
	Always	17 (26.6%)	4 (6.3%)	11 (30.6%)	1 (2.8%)
How do you rate your level of sexual desire?	Very High	0 (0.0%)	19 (29.7%)	0 (0.0%)	6 (16.7%)
	High	9 (14.1%)	7 (10.9%)	5 (13.9%)	0 (0.0%)
	Moderate	34 (53.1%)	23 (35.9%)	16 (44.4%)	22 (61.1%)
	Low	17 (26.6%)	7 (10.9%)	11 (30.6%)	1 (2.8%)
	Very Low	4 (6.3%)	8 (12.5%)	4 (11.1%)	1 (2.8%)
How many times do you feel pain during sexual intercourse?	Never	30(46.9%)	22 (34.4%)	14 (38.9%)	17 (47.2%)
	Rarely	32 (50.0%)	2 (3.1%)	19 (52.8%)	1 (2.8%)
	Sometimes	2 (3.1%)	16 (25.0%)	3 (8.3%)	14 (38.9%)
	Usually	0 (0.0%)	6 (9.4%)	0 (0.0%)	2 (5.6%)
	Always	0 (0.0%)	18 (28.1%)	0 (0.0%)	2 (5.6%)

For other cosmetic procedures, “feeling aroused during the sexual intercourse” was reported as “rarely or never” in 66.6% before the procedures and in 33.3% of the women after the procedures. Feeling fulfilled during sexual intercourse was reported as “rarely or never” in 86.1% before the procedures and in 36.2% after the procedures. Feeling shame during sexual intercourse was reported as “rarely or never” by 11.1% before the procedures and by 80.6% after the procedures. Feeling fear during sexual intercourse was reported as “rarely or never” in 25% before the procedures and in 88.9% after the procedures. The level of sexual desire reported as “very high or high” increased from 13.9% before to 16.7% after the procedures. Feeling pain during sexual intercourse was reported as “rarely or never” in 91.7% before the procedures and in 50% after the procedures.

Table 4 presents the different procedure types in the context of Satisfaction Rate/Quality of Life after Procedure; participants who underwent vaginoplasty were satisfied, and the QoL improvement was at 82.1%. Participants who underwent labiaplasty were extremely satisfied and happy; their satisfaction rate was 90.1%. However, the satisfaction rate was only 75% in participants who underwent augmentation of labia majora by filler. While 95.9% of participants who underwent liposuction and/or fat transfer were not satisfied at all and did not report an improvement in their QoL, those who underwent multiple combined procedures reported that they were very satisfied and also reported a significant improvement in QoL.

**Table 4.** Types of Procedures in Comparison With Satisfaction Rate/Quality of Life After Procedure

Procedure Type	Count (Total *N* %)	Improvement of Satisfaction Rate/ Self-esteem/ QoL
Vaginoplasty	(39) 39.0%	Yes (32) 82.1% No (7) 17.9%
Labiaplasty (labia minora reduction)	(2) 22.0%	Yes (20) 90.9% No (2) 9.1%
Labiaplasty (labia majora reduction)	(2) 2.0%	Yes (20) 90.9% No (2) 9.1%
Augmentation of labia majora by filler	(8) 8.0%	Yes (6) 75.0% No (2) 25.0%
Liposuction and fat transfer	(1) 1.0%	Yes (0) 0.0% No (1) 100.0%
Multiple combined procedures	(28) 28.0%	Yes (27) 95.9% No (1) 4.1%

QoL, quality of life.

Overall, 86% of the women noticed an improvement in their satisfaction rate and self-esteem after the procedures, while 14% did not.

## DISCUSSION

FCGS has been increasingly requested by women. Women who underwent genital aesthetic surgery due to sexual dissatisfaction showed an improvement in genital appearance as well as in sexual function.^9^ The first paper on labiaplasty was published in 1976; from 1976 to the current date, approximately 25 papers have been published on this subject.^10^ Our study indicates that FCGS improves the external appearance of genitals. According to Sharp et al,^11^ after labiaplasty, most of the women were satisfied with the outcomes of genital appearance, psychological well-being, and sexual function. However, a study from 2016 reported no differences in the measures of psychological well-being and relationship quality between women who underwent labiaplasty and those who did not. In addition, women who underwent labiaplasty were less satisfied.^3^ In our study, we found that the satisfaction rate and psychological well-being (self-confidence and self-esteem improvement) were substantially increased by 50% and 40%, respectively, while feelings of embarrassment during sexual intercourse decreased by 36.1%. Patients who underwent labiaplasty and/or vaginoplasty were satisfied and reported a significant improvement in genital appearance and psychological parameters. According to Furnas,^12^ the positive consequence of labiaplasty depends on the technique employed. For instance, the most commonly used technique is the wedge, which is an excellent choice for women with skinny, well-marked labial edges and for those dissatisfied with their thick, rough, or dark labial edges. Aesthetic surgeries are currently being used for enhancing women’s self-esteem and self-satisfaction.^6^ For the labia minora reduction procedure, after drawing the desired shape, a linear incision was made using electrocautery; a continuous running polyglactin 910 suture was then used. The labia majora reduction was performed by making an elliptical vertical incision in the labia majora; the minora/majora intersection was closed and approximated in 3 layers to reduce the risk of dehiscence. The global complication rate of labiaplasty is 13%; the most common complication of labia minora reduction is wound dehiscence, which is more likely to develop in cases of wedge resections rather than in cases of edge resection.^13^ According to Gowda et al,^14^ the most common complications caused by labia minora reduction are wound dehiscence, hematoma, scarring, and infection, and also flap necrosis in the case of wedge resection. Additionally, a systematic review of labiaplasty has reported the following: 5 papers did not assess complications; 8 papers denied the presence of complications; and 6 papers documented complications, including infection, bleeding, and wound dehiscence. Three papers stated that wound dehiscence was minor and did not require re-suturing or repeat surgery. The majority of patients who underwent labiaplasty were aged between 16 and 35 years. The overall outcome of labiaplasty was good, the patients were satisfied, and there was a marked improvement in appearance and psychological measures.^10^ In our study, the complication rate was low (8%), and none of the participants needed repeat surgery. Hence, the procedures can be termed as safe and effective. According to Barbara et al,^2^ vaginoplasty (or vaginal reconstruction) is considered as a standard surgical technique and is a safe and effective procedure for improving sexual function in women who suffer from an acquired sensation of vaginal laxity and related sexual dissatisfaction. A US survey related to the American Society of Plastic Surgeons reported an elevation in the prevalence of vaginal rejuvenation procedures from 793 in 2005 to 1030 in 2006 (30% increase), and the majority of these procedures were performed to improve appearance, sexual function, and vaginal tone.^2^ The ideal vaginoplasty method should give excellent aesthetic and functional results, with a low rate of complications.^15^ In our study, a v-shaped perineal incision was first made, followed by a midline posterior vaginal wall incision up to 1–2 cm from the cervix. The vaginal epithelium was reflected from the underlying tissues; the excess epithelium was trimmed off; and a no. 0 polyglactin 910 suture was used to enforce the perineal muscles. The vaginal epithelium was approximated using a continuous running 2-0 polyglactin 910 suture.

In this study, the majority of women (64%) who underwent vaginoplasty reported improved self-esteem and satisfaction rates; psychological measures improved by approximately 40%. Interestingly, we detected that the concern of the women about the “appearance of genitals,” “looks normal in appearance,” and “feeling genitals unattractive” decreased by 35.5% after vaginoplasty. In our study, PISQ scores for “feeling aroused,” “feeling fulfilled during sexual intercourse,” and “level of sexual desire” increased after vaginoplasty, while those for “feeling of shame or fear during sexual intercourse” decreased. However, it is important to note that feelings of pain during sexual intercourse were crucially increased after this procedure. Thus, proper presurgery counseling has provided, which includes a discussion of persistent pain after vaginoplasty.

The other cosmetic procedures resulted in significant improvements in psychological measures and self-confidence, as evidenced by increased “feeling aroused and fulfilled during sexual intercourse” and decreased “feeling of shame or fear during sexual intercourse.” However, an increase in the level of sexual desire and pain during sexual intercourse was reported. Thus, we found an improvement in the Sexual Function Questionnaire results. A study of FCGS published in 2019 documented a successful outcome with a high satisfaction rate.^16^ In line with the above, the results of our study showed an overall improvement of 86% for the satisfaction rate, which is considered to be an excellent outcome. Multiple combined procedures were most effective and resulted in a very high satisfaction rate and markedly increased QoL and self-esteem. Liposuction and/or fat transfer were relatively ineffective because the satisfaction rate was low; however, because only one participant underwent this procedure, this result is unreliable. In addition, no improvements were reported in self-esteem and the QoL.

It is important to note that some patients requesting FCGS may have a history of mood disorders, persistent distress thoughts, or mental disorders such as body dysmorphic disorder (BDD), as documented in other cosmetic surgery patients. According to the *Diagnostic and Statistical Manual of Mental Disorders*, 5th edition (DSM-5), BDD is defined as an intense preoccupation with minor or illusory defects in physical appearance associated with interfering thoughts, persistent distress, significant impairment in social and occupational functioning, and repetitive behaviors, such as mirror-checking, seeking reassurance from others, and requesting unnecessary cosmetic surgery.^17^ The Cosmetic Procedure Screening (COPS) Questionnaire is a brief questionnaire used for screening and identifying people with BDD. However, the questionnaire includes queries about BDD diagnostic criteria, features/concerns that the patient finds unattractive, and the type of the cosmetic procedures they are seeking. It comprises of 9 items, which are scored from 0 (least impaired) to 8 (most impaired).^18^

The study has several important limitations. First, the sample size was small and may not have been representative of the general population of women who usually seek FCGS. Moreover, these women were sourced from 2 private clinics in one city. Second, the possibility of recall bias that may have impacted the results, and third, a response rate of only 51%; the low response rate may be because many women did not answer the phone when called either because the number was unknown to them or because they had changed their phone numbers.

Nevertheless, this study has some strengths. This is one of the first studies to examine the factors that motivate women to undergo FCGS in the region, providing new insights into the associated motivations and psychological aspects; this knowledge can be used as a springboard for future research.

As expected, FCGS is popular among women of Saudi Arabia and the Middle East; still, there is a dearth of labiaplasty and vaginal rejuvenation prevalence studies from these regions.

## CONCLUSIONS

FCGSs are effective surgical procedures, and the majority of women who underwent genital aesthetic surgery of the labia minora and majora and vaginoplasty for both aesthetic and functional causes reported satisfaction; psychological measures improved as well. However, it is important to note that pain during sexual intercourse worsened. Furthermore, the low rate of postsurgical complications indicates that cosmetic genital procedures are safe overall.

## Supplementary Material

ojaa048_suppl_Supplementary_Appendix-A

ojaa048_suppl_Supplementary_Appendix-B

## References

[1] Magon N, Alinsod R. Female cosmetic genital surgery: delivering what women want. J Obstet Gynaecol India. 2017;67(1):15-19. 28242962 10.1007/s13224-016-0930-y PMC5306104

[2] Barbara G, Facchin F, Buggio L, Alberico D, Frattaruolo MP, Kustermann A. Vaginal rejuvenation: current perspectives. Int J Womens Health. 2017;9:513-519. 28860864 10.2147/IJWH.S99700 PMC5560421

[3] Sharp G, Tiggemann M, Mattiske J. Factors that influence the decision undergo labiaplasty: media, relationships, and psychological well-being. Aesthet Surg J. 2016;36(4):469-478. 26893523 10.1093/asj/sjv270

[4] Surroca MM, Miranda LS, Ruiz JB. Labiaplasty: a 24-month experience in 58 patients: outcomes and statistical analysis. Ann Plast Surg. 2018;80(4):316-322. 29461293 10.1097/SAP.0000000000001395

[5] Tepper OM, Wulkan M, Matarasso A. Labioplasty: anatomy, etiology, and a new surgical approach. Aesthet Surg J. 2011;31(5):511-518. 21719863 10.1177/1090820X11411578

[6] Goodman MP, Placik OJ, Benson RH 3rd, et al. A large multicenter outcome study of female genital plastic surgery. J Sex Med. 2010;7(4 Pt 1):1565-1577. 19912495 10.1111/j.1743-6109.2009.01573.x

[7] Veale D, Eshkevari E, Ellison N, Cardozo L, Robinson D, Kavouni A. Validation of genital appearance satisfaction scale and the cosmetic procedure screening scale for women seeking abiaplasty. J Psychosom Obstet Gynaecol. 2013;34(1):46-52. 23394414 10.3109/0167482X.2012.756865

[8] Al-Badr A, Al-Juraifani R, Al-Sheikh G, Al-Mandeel H, Al-Dakhyel L, Al-Shahrani N. Validation of the International Urogynecology Association’s pelvic organ prolapse/urinary incontinence sexual questionnaire in Arabic. Int Urogynecol J. 2017;28(3):437-445. 27678143 10.1007/s00192-016-3160-z

[9] Goodman MP, Placik OJ, Matlock DL, et al. Evaluation of body image and sexual satisfaction in women undergoing female genital plastic/cosmetic surgery. Aesthet Surg J. 2016;36(9):1048-1057. 27084062 10.1093/asj/sjw061

[10] Liao LM, Michala L, Creighton SM. Labial surgery for well women: a review of the literature. BJOG. 2010;117(1):20-25. 19906048 10.1111/j.1471-0528.2009.02426.x

[11] Sharp G, Tiggemann M, Mattiske J. A retrospective study of the psychological outcomes of labiaplasty. Aesthet Surg J. 2017;37(3):324-331. 28207030 10.1093/asj/sjw190

[12] Furnas HJ . Trim labiaplasty. Plast Reconstr Surg Glob Open. 2017;5(5):e1349. 28607869 10.1097/GOX.0000000000001349 PMC5459652

[13] Ouar N, Guillier D, Moris V, Revol M, Francois C, Cristofari S. [Postoperative complications of labia minora reduction. Comparative study between wedge and edge resection]. Ann Chir Plast Esthet 2017;62(3):219-223. 28285885 10.1016/j.anplas.2017.02.005

[14] Gowda AU, Chopra N, Khalifeh M. Indications, techniques and complications of labiaplasty. Eplasty. 2015;15:ic46. 26330896 PMC4544281

[15] Motta GL, Tavares PM, Silva GVM, Berger M, Neto SB, Rosito TE. Full-thickness skin mesh graft vaginoplasty: a skin sparing technique. Int Braz J Urol. 2017;43(6): 1193. 28191788 10.1590/S1677-5538.IBJU.2016.0259 PMC5734087

[16] Kalaaji A, Dreyer S, Maric I, Schnegg J, Jönsson V. Female cosmetic genital surgery: patient characteristics, motivation, and satisfaction. Aesthet Surg J. 2019;39(12):1455-1466. 30423019 10.1093/asj/sjy309

[17] Barbara G, Facchin F, Meschia M, Vercellini P. “The first cut is the deepest”: a psychological, sexological and gynecological perspective on female genital cosmetic surgery. Acta Obstet Gynecol Scand. 2015;94(9):915-920. 25891185 10.1111/aogs.12660

[18] Veale D, Ellison N, Werner TG, Dodhia R, Serfaty MA, Clarke A. Development of a Cosmetic Procedure Screening Questionnaire (COPS) for body dysmorphic disorder. J Plast Reconstr Aesthet Surg. 2012;65(4):530-532. 22000332 10.1016/j.bjps.2011.09.007

