# Supplementary material for: Satisfaction Survey of Women After Cosmetic Genital Procedures: A Cross-Sectional Study From Saudi Arabia
Source: Aesthet Surg J Open Forum. 2020 Nov 10;3(1):ojaa048. doi: 10.1093/asjof/ojaa048 (PMC7750879; doi:10.1093/asjof/ojaa048)
Supplement: ojaa048_suppl_Supplementary_Appendix-A [file ojaa048_suppl_supplementary_appendix-a.pdf]

(Satisfaction Rate among Saudi Women Toward Vaginal cosmetic surgeries : Vaginoplasty, Labiaplasty, Its Effect on Their Sexual Functions and Daily Life)

ALL THE INFORMATION IS CONFIDENTIAL FOR THE RESEARCHERS

Personal Data:

- Age:………………..
- Weight:…………..
- Height:…………….
- Number of Children:………..
- Educational Level:……………
- Procedure Date:………………

Procedure & Result:

- What are the motivations to undergone the procedure:
  - Didn't like the appearance
  - Discomfort when walking, sitting or doing activities
  - Discomfort during or after sexual intercourse
  - Decrease self-esteem and confidence.

- Procedure Type: ……………………………
- Do you notice improvement in your life after procedure:
  -Yes -No
- Were there complications, another surgery?...............................

- Genital Appearance Satisfaction Scale (GAS) :
  <u>BEFORE SURGERY</u>

1. I feel that my genital are normal:
   Strongly agree somewhat agree somewhat disagree strongly disagree

2. I feel that my genital are unattractive in appearance :
   Strongly agree somewhat agree somewhat disagree strongly disagree

3. I feel that my labia are too large :
   Strongly agree somewhat agree somewhat disagree strongly disagree

4. I am satisfied with the appearance of my genitals :
   Strongly agree somewhat agree somewhat disagree strongly disagree

5. I experience irritation to my labia when walking/exercising :
   Strongly agree somewhat agree somewhat disagree strongly disagree

6. I feel conscious in sexual situations because the appearance of my genitals :
   Strongly agree somewhat agree somewhat disagree strongly disagree

7. Embarrassment of my genitals spoils the enjoyment of my sex :
   Strongly agree somewhat agree somewhat disagree strongly disagree

8. I feel that my genitals are appear with tight clothes :
   Strongly agree somewhat agree somewhat disagree strongly disagree

9. I worry about the appearance of my vaginal area :
   Strongly agree somewhat agree somewhat disagree strongly disagree

10-I feel that my genitals are asymmetric :
Strongly agree somewhat agree somewhat disagree strongly disagree

11- Satisfaction rate before surgery :

5	4	3	2	1

12- Improvement in psychological aspect :

5	4	3	2	1

13-Improvement in self-esteem and self-confidence:

5	4	3	2	1

- Genital Appearance Satisfaction Scale (GAS) :
  AFTER SURGERY

1-I feel that my genital are normal:

Strongly agree somewhat agree somewhat disagree strongly disagree

2-I feel that my genital are unattractive in appearance :

Strongly agree somewhat agree somewhat disagree strongly disagree

3-I feel that my labia are too large :

Strongly agree somewhat agree somewhat disagree strongly disagree

4-I am satisfied with the appearance of my genitals :

Strongly agree somewhat agree somewhat disagree strongly disagree

5-I experience irritation to my labia when walking/exercising :

Strongly agree somewhat agree somewhat disagree strongly disagree

6- I feel conscious in sexual situations because the appearance of my genitals :

Strongly agree somewhat agree somewhat disagree strongly disagree

7-Embarrassment of my genitals spoils the enjoyment of my sex :

Strongly agree somewhat agree somewhat disagree strongly disagree

8-I feel that my genitals are appear with tight clothes :

Strongly agree somewhat agree somewhat disagree strongly disagree

9-I worry about the appearance of my vaginal area :

Strongly agree somewhat agree somewhat disagree strongly disagree

10-I feel that my genitals are asymmetric :

Strongly agree somewhat agree somewhat disagree strongly disagree

11- Satisfaction rate before surgery :

5	4	3	2	1

12- Improvement in psychological aspect :

5	4	3	2	1

13-Improvement in self-esteem and self-confidence:

5	4	3	2	1

- Pelvic organ prolapse/ urinary incontinence and sexual questionnaire : <u>SEXUAL ACTIVITY</u>

1- How often do you feel aroused during sexual activity:
Never Rarely Sometimes Usually Almost Always

2- When you involve in sexual activity do you feel fulfilled:
Never Rarely Sometimes Usually Almost Always

3- When you involve in sexual activity do you feel shame:
Never Rarely Sometimes Usually Almost Always

4-When you involve in sexual activity do you feel fear:
Never Rarely Sometimes Usually Almost Always

5-How rate the level of sexual desire now:
Never Rarely Sometimes Usually Almost Always

6- How rate the level of sexual desire now:
Never Rarely Sometimes Usually Almost Always

7-How many time you feel pain during sexual intercourse:
Never Rarely Sometimes Usually Almost Always
