# Supplementary material for: Satisfaction Survey of Women After Cosmetic Genital Procedures: A Cross-Sectional Study From Saudi Arabia
Source: Aesthet Surg J Open Forum. 2020 Nov 10;3(1):ojaa048. doi: 10.1093/asjof/ojaa048 (PMC7750879; doi:10.1093/asjof/ojaa048)
Supplement: ojaa048_suppl_Supplementary_Appendix-B [file ojaa048_suppl_supplementary_appendix-b.pdf]

(قياس مستوى الرضى لدى النساء تجاه تجميل المهبل أو الشفرات وتأثيرها على حياتهم اليومية والجنسية)

جميع المعلومات محفوظة بسريّة تامة لدى الباحثين

البيانات الشخصية:

- العمر: ---------------------
- الوزن:---------------------
- الطول:----------------------
- عدد الأطفال :----------------------
- المستوى التعليمي :------------------------
- تاريخ إجراء العملية :---------------------------

الإجراء والنتائج :

- ماهي الأسباب التي دفعتك لإجراء عملية تجميل الشفرات :
  - الإنزعاج من المظهر الخارجي
  - عدم الراحة أثناء ممارسة الرياضة أو إرتداء بعض الملابس
  - الإنزعاج خلال أو بعد الممارسة الجنسية
  - الإنزعاج النفسي وإنعدام الثقه

- نوع/أسم الاجراء :--------------------------
- هل تشعرين ان هناك تحسن في طبيعة حياتك بعد إجراء عمليه تجميل الشفرات :----------------------
- هل هناك أي مضاعفات أو إجراء عملية أخرى لتعديل العملية الأولى:---------------------------

مقياس مستوى الرضى على شكل العضو التناسلي قبل وبعد إجراء العملية :

قبل إجراء العملية :

- هل تشعرين أن شكل العضو التناسلي طبيعي :

- أوافق بشده -اوافق احيانا - لا اوافق احيانا - لا أوافق بشده

- هل تشعرين أن العضو التناسلي كان غير جذاب:

-أوافق بشده -اوافق احيانا - لا اوافق احيانا - لا أوافق بشده

- هل تشعرين أن الشفره الصغرى او الكبرى كبيره في الحجم:

- أوافق بشده -اوافق احيانا - لا اوافق احيانا - لا أوافق بشده

- مستوى شعورك بالرضى:

1 (مرضية)	2	3	4	5(غيرمرضية)

- مدى التأثير من الناحيه النفسية قبل العملية :

1 (مرضية)	2	3	4	5(غيرمرضية)

- مدى التحسن من ناحية الثقة بالنفس قبل العملية :

1 (مرضية)	2	3	4	5(غيرمرضية)

- هل كان هناك أي تهيج او شعور بعدم الراحه خلال ممارسة التمارين الرياضيه او المشي:

- نعم -لا

- هل شعرتي بالاحراج بسبب شكل العضو التناسلي أثناء الجماع:

- نعم -لا

- هل تشعرين بعدم الراحه عند ارتداء الملابس الضيقه بسبب العضو التناسلي:

- نعم -لا

- هل تشعرين ان منطقة العضو التناسلي بارزه عند ارتداء الملابس الضيقه:

- نعم -لا

- هل تشعرين بالتوتر من شكل العضو التناسلي :

- نعم -لا

- هل تشعرين أن الشفرتين الصغرى او الكبرى غير متماثلتان :

- نعم -لا

مقياس مستوى الرضى على شكل العضو التناسلى قبل وبعد إجراء العملية :

بعد إجراء العملية :

- هل تشعرين أن شكل العضو التناسلي طبيعي :
  - أوافق بشده -اوافق احيانا - لا اوافق احيانا - لا أوافق بشده وافق - لا أوافق
- هل تشعرين أن العضو التناسلي أصبح غير جذاب :
  -أوافق بشده -اوافق احيانا - لا اوافق احيانا - لا أوافق بشده
- هل تشعرين أن الشفره الصغرى او الكبرى كبيره في الحجم :
  -أوافق بشده -اوافق احيانا - لا اوافق احيانا - لا أوافق بشده
- مستوى شعورك بالرضى :

1 (مرضية)	2	3	4	5(غيرمرضية)

- مدى التحسن من الناحيه النفسية :

1 (مرضية)	2	3	4	5(غيرمرضية)

- مدى التحسن من ناحية الثقة بالنفس :

1 (مرضية)	2	3	4	5(غيرمرضية)

- هل كان هناك أي تهيج او شعور بعدم الراحه خلال ممارسة التمارين الرياضيه او المشي :
  - نعم -لا
- هل شعرتي بالإحراج بسبب شكل العضو التناسلي أثناء الجماع :
  - نعم -لا
- هل تشعرين بعدم الراحه عند إرتداء الملابس الضيقه بسبب العضو التناسلي :
  - نعم -لا
- هل تشعرين أن منطقة العضو التناسلي بارزه عند إرتداء الملابس الضيقه :
  - نعم -لا
- هل تشعرين بالتوتر من شكل العضو التناسلي :
  - نعم -لا
- هل تشعرين أن الشفرتين الصغرى أو الكبرى غير متماثلتان :
  - نعم -لا

النشاط الجنسي :

- ما مدى شعورك بأنك مستثارة جنسيا (مستثارة جسديا أو متحمسة)خلال النشاط الجنسي؟

أبداً	نادراً	أحياناً	عادةً	دائماً

- أثناء العالقة الجنسية (الجماع) كم مره تشعرين بالأتي:
  - التشبع

أبداً	نادراً	أحياناً	عادةً	في الغالب

  - الخجل أو الحرج

أبداً	نادراً	أحياناً	عادةً	في الغالب

  - الخوف

أبداً	نادراً	أحياناً	عادةً	في الغالب

- مقارنة بنشوتك الجنسية (أي الوصول للرضى، أو قمة النشوة أو نهاية المتعه) في السابق، مامدى شدة نشوتك الجنسية الأن؟

أقل بكثير	أقل	نفس القوة	أقوى	أقوى بكثير

- كم مرة تشعرين بالألم خلال الجماع

أبداً	نادراً	احياناً	عادةً	دائماً
